# Attitudes, Beliefs and Predictors of Male Circumcision Promotion among Medical University Students in a Traditionally Non-Circumcising Region

**DOI:** 10.3390/ijerph14101097

**Published:** 2017-09-21

**Authors:** Maria Ganczak, Marcin Korzeń, Maciej Olszewski

**Affiliations:** 1 Department of Epidemiology and Management, Faculty of Medical Sciences, Pomeranian Medical University, 71-210 Szczecin, Poland; 2 Department of Methods of Artificial Intelligence and Applied Mathematics, Faculty of Computer Science and Information Technology, West Pomeranian University of Technology, 71-210 Szczecin, Poland; mkorzen@wi.zut.edu.pl; 3 Students’ Scientific Association, Pomeranian Medical University, 70-204 Szczecin, Poland; olschevski@gmail.com

**Keywords:** male circumcision, attitudes, beliefs, determinants, promotion, medical students

## Abstract

*Objective*: To evaluate the beliefs of medical university students regarding male circumcision (MC), as well as attitudes and the predictors of its promotion in the case of adults at risk of HIV. *Methods*: A cross-sectional survey was conducted between 2013–2016 at the Medical University in Szczecin, Poland, among final year Polish/foreign students from Northern Europe, using a standardized questionnaire. *Results*: There were 539 participants, median age 25 years, 40.8% males, and 66.8% were Polish nationals. The MC rate was 16.7%. Regarding HIV/AIDS knowledge, 66.6% of the students scored more than 75%; and, 34.2% knew that MC reduces the risk of HIV infection. One in eleven respondents (9.1%) believed that circumcised men felt more intense sexual pleasure. More than half of the respondents (54.8%) declared that they would recommend MC to adult patients at risk for HIV. The belief that circumcised men felt more intense sexual pleasure, and knowledge on MC regarding HIV risk reduction was associated with greater odds of recommending adult MC (OR = 3.35 and OR = 2.13, respectively). *Conclusions*: Poor knowledge of its benefits and a low willingness to promote the procedure—strongly dependent on personal beliefs—suggest that medical students may need additional training to help them to discuss MC more openly with adult men at risk for HIV infection. Knowledge may be an effective tool when making decisions regarding MC promotion.

## 1. Introduction

It is important to tackle HIV transmission by using strategies that may improve population health; and this includes male circumcision (MC). Three large randomized controlled trials (RCTs) conducted in African countries have shown that MC reduces the risk of acquiring HIV through heterosexual intercourse in males by 51–60% [1], and has been associated with a decreased risk of acquiring human papillomavirus, syphilis, and urinary tract infections [2,3]. These findings are currently being taken into account with regards to health recommendations and policy proposals. In areas with high endemic rates of HIV, the World Health Organization and Joint United Nations Programme on HIV and AIDS recommend MC as a part of a comprehensive program for prevention of HIV transmission. MC is a procedure that can be performed not only during infancy, but also in adolescence or adulthood [4]. As such, it could be discussed with those adults at risk of contracting HIV [1]. 

However, the inclusion of MC in the health policy of developed countries with low endemic HIV rates, such as those in Europe, requires further scrutiny [5]. Currently, no major medical organization has either recommended or banned universal neonatal circumcision in these regions. In 2012, the American Academy of Pediatrics (APP) and the American Congress of Obstetricians and Gynecologists published recommendations stating that doctors should discuss the benefits and risks of newborn MC with parents [6]. This stance is very similar to that of the Centers for Disease Control and Prevention, the Canadian Medical Society, the British Medical Association, the Royal Australasian College of Physicians, and the Circumcision Academy of Australia [7,8]. These policy statements have arisen in the context of a growing body of evidence supporting the medical benefits of MC. Despite this evidence, MC rates in developed countries have been in decline in recent years [9]. 

In some European countries, especially Poland, negative attitudes towards MC persist, largely stemming from the period of Nazi occupation during World War II, when MC was perceived as Judaism-related and possibly provoking repression and even leading to a death sentence [10]. 

Thus, MC promotion in the region remains challenging. MC opponents in Europe may also point to its minimal anticipated impact, since HIV rates per 100,000 of the population [11] are much lower than in some African countries [12], where the overall risk regarding HIV transmission is very high. 

In Europe, males are reported as the more affected gender. In Poland, in 2014, almost two thirds of newly diagnosed infections among men with known transmission category were acquired by men who had sex with other men (MSM). However, no increase has been observed in the number of cases in this population group as compared to 2013 [13]. There has been a 32% increase among people infected through heterosexual contact. Sex between men accounted for more than half of new diagnoses in 14 other European countries (Austria, Croatia, Cyprus, the Czech Republic, Germany, Greece, Hungary, Ireland, Malta, the Netherlands, Slovakia, Slovenia, Spain, and the United Kingdom). The prevalence rates of new HIV cases per 100,000 of the population in those countries varied between 1.6 in Slovakia and 9.6 in the UK. In Scandinavia, the prevalence rates were low (3.3–5.2), although higher than that observed in Poland (2.8). However, a different pattern regarding the transmission mode was reported, with heterosexual transmission being the most commonly reported known mode in Finland, Norway, and Sweden. 

Before effective interventions to promote MC can be introduced on a national scale in traditionally non-circumcising European regions, thorough studies need to be conducted. MC rates and its cost-effectiveness need to be analyzed in order to determine whether the policy of promoting and providing inexpensive access to voluntary circumcision for adult men would indeed result in lower overall societal healthcare costs [14]. Of note, in Poland, MC performed for non-medical reasons has to be covered by the patient and costs on average, around 260–400 USD. The cost of medical MC is paid for through health insurance. It is estimated that about 13,500 procedures are performed annually, mainly to treat pathological phimosis and refractory balanoposthitis [15].

Another obstacle regarding adult MC promotion might be a reluctance to accept circumcision as an HIV preventative measure by communities that do not traditionally circumcise. Therefore, to study predictors regarding the acceptability of this practice among health care providers and patients would be also of value.

## 2. Objectives

The objective of the study was to assess MC prevalence in a traditionally non-circumcising region, and to evaluate the beliefs of medical university students regarding male circumcision (MC), as well as attitudes, and the predictors of its promotion in the case of adults at risk of HIV. The main goal was to assess the potential for introducing culturally applicable interventions tailored to promote MC as an element of HIV prevention strategy. To our knowledge, similar studies on MC related to European medical students, as prospective health care providers, have not yet been carried out.

## 3. Materials and Methods

### 3.1. Design, Setting, Population and Sampling

This was a cross-sectional survey, conducted between 2013–2016 at a national government university (The Pomeranian Medical University) situated in Szczecin, in the north west of Poland. The study population consisted of 541 final year medical students who were enrolled in the program—Poles from different regions of the country, as well as foreign students from Scandinavia and Germany, attending a medical course taught in English. All of the students who were present at the time when the questionnaires were administered were asked to participate. As part of the consenting process, it was made clear that there would be no penalty for refusing to participate in this study.

### 3.2. Data Collection 

One research team member (Maciej Olszewski) administered the questionnaires under normal classroom conditions. A structured, anonymous questionnaire, with separate versions for males and females was adapted for local conditions from a questionnaire used in a previous research addressing MC by Westercamp et al. [16]. The authors obtained consent to use the original questionnaire and it was then translated for Polish respondents. Participants were asked about:

(i) demographic data (age, gender, nationality, religion, and circumcision status in the case of male participants);
(ii) sources of knowledge with regard to MC;
(iii) knowledge of HIV/AIDS regarding transmission modes; clinical manifestations; testing; treatment; and, preventive measures;
(iv) knowledge about MC regarding the risk of HIV transmission, determined by asking participants how likely a circumcised man would become infected with HIV as compared to an uncircumcised individual;
(v) if they knew someone who had been circumcised; and,
(vi) beliefs about MC measured by asking participants if, in their opinion: (1) circumcised men feel more intense/about the same/less sexual pleasure than uncircumcised men, (2) penis sensitivity decreases/ increases/remains the same after MC, and (3) is MC a safe procedure.

Knowledge of HIV/AIDS was assessed by giving 1 point for each correct answer to the 12 items rated as “true”, “false”, “don’t know”. The scale measured knowledge from a minimum of 0 to a maximum of 12. Scores for individuals were summed up to give a total knowledge score. Scores of 0–5 (less than 50% answered correctly) were arbitrary taken as poor, 6–9 (50–75% correct answers)—as mediocre knowledge, 10–12 (more than 75% correct answers)—as good knowledge. 

Circumcised males were asked about their age at circumcision and the primary reason for the procedure.

The main question of interest, developed for the purpose of this study, was: “When counseling a male at risk of HIV infection, would you generally recommend adult circumcision?”. The possible answers were (1) yes, (2) no, (3) not sure. Heterosexual men at an increased risk for HIV included those who are in sexual relationships with HIV infected female partners, men with multiple female partners, those in relationships with women who are at high risk of HIV (e.g., commercial sex workers, intravenous drug users). The study was approved by the Pomeranian Medical University Ethics Committee (KB 0012/41/13). 

### 3.3. Statistical Analysis 

Data analysis was carried out using STATISTICA PL Version 12.5 (StatSoft, Polska, Kraków, Poland) and R software (Lucent Technologies, New providence, NJ, USA) [17]. Bivariate analysis examined demographic and other characteristics: age (<25/≥25years), gender, nationality (Polish/other), religion (Roman Catholic—other Christian—Muslim/atheist), being circumcised (yes/no; analysis referred to males only), knowing a circumcised male (yes/no), participant beliefs in MC (such as: MC is a safe procedure: yes/no–not sure; circumcised men feel more intense sexual pleasure than uncircumcised men/less intense—the same; MC increases penis sensitivity/decreases —no effect), knowledge regarding HIV/AIDS (good/mediocre-poor), and knowledge of MC regarding the impact on HIV risk (it is less likely circumcised men become infected with HIV/more likely—don’t know), associated with an outcome variable. For categoric variables, groups were compared using the chi-square test with Yates correction and Fisher’s exact test; the Mann-Whitney test was used for numeric variables. For the predicted outcome variable listed above, a standard single-outcome logistic model was built; the model was reduced with the use of a stepwise selection [18]. For main regression analyses, the three possible responses were dichotomized into two categories: willing (yes) or not willing (no—not sure). Regression coefficients in the regression model were used to assess any change in the model. A change in coefficients was compared and used to determine any variable change.

## 4. Results

### 4.1. Demographics

The response rate was 99.6%, with 539 students (Me of age 25 years, range 22–38 years) participating. Males constituted 40.8% of the sample, Polish nationals—66.8%. The vast majority of students (79.7%) declared being religious: more than half (59.0%) were Roman Catholics, 16.7%—other Christians, 4.5%—Muslims; 19.9% were atheists (Table 1). There were no statistically significant between-gender differences regarding age (*p* = 0.89), nationalities (*p* = 0.36 and *p* = 0.98, respectively, regarding Polish and other students), and religion (*p* = 0.91, *p* = 0.98 and *p* = 0.29 respectively regarding Roman Catholics, other Christians, and atheists). There were significantly more males among Muslims than females (*p* = 0.02).

### 4.2. Circumcision Rates

Among the 192 men who answered the question, 32 (16.7%) reported being circumcised (Table 1); the percentage was lower among the Polish students as compared to other nationalities (4.6% vs. 42.6%, respectively), *p* < 0.0001. The median of reported age at circumcision was 1.5 years (range 0–26 years). All of the Polish students were circumcised for medical reasons, 61.5% foreign students were circumcised for religious reasons. More than one fourth of participants (29.7%) knew someone who had been circumcised.

### 4.3. Sources of MC Information

Most frequently students had obtained information on MC from university staff (66.1%), media-radio/TV/newspapers (60.2%), medical personnel (29.4%), and an individual who had been circumcised (28.0%); this was a multiple-choice question.

### 4.4. Beliefs of MC 

Of the 343/539 (63.6%) respondents who answered this question, 295 (86.0%) stated that MC is a safe procedure, 28 (8.2%) answered that it is not safe, 20 (5.8%) were not sure. In terms of the impact on sexual satisfaction, of the 471 participants (87.4%) who responded to this question, 9.1% stated that circumcised men feel more intense sexual pleasure than uncircumcised men, 25.7%—that they feel less intense pleasure, 25.5%—about the same, 39.7%—were unsure. Of the 475 (88.1%) participants who responded to the statement “MC changes the sensitivity of the penis”, 32.2% stated that it decreases sensitivity, 17.5%—that it increases sensitivity, 15.8%—that sensitivity remains about the same, 34.5%—were unsure. Answers to those two questions were correlated (Kendall’s tau-b coefficient r = 0.27, *p* < 0.001).

### 4.5. Knowledge on HIV/AIDS 

The mean score of HIV/AIDS knowledge was 9.49, SD ± 2.2. Two-thirds (n = 359; 66.6%) of the respondents scored more than 75% correct answers, 28 (5.2%)—less than 50%. The majority of students (96.1%) correctly identified mother-to-child transmission (MTCT) as a possible route of HIV transmission, as shown in Table 2. Vaginal receptive intercourse was correctly recognized as not the highest risk in terms of contracting HIV infection by 58.0% of the respondents. The majority of participants (87.4%) were aware that most people with HIV did not show signs of being sick in the first week after infection; and, 71.4% correctly interpreted a positive blood test result. Regarding HIV treatment, 94.8% of respondents had heard about anti-retroviral therapy (ART), the vast majority (71.2–89.4%) correctly answered ART-related questions; only 18.0% correctly marked that globally, ART was not available to almost all of those who needed it. In the overall sample, 95.2% recognized using a condom as a protective measure to prevent HIV infection. 

### 4.6. Knowledge on the Association between MC and the Risk of Contracting HIV

Participants were asked how likely circumcised men become infected with HIV as compared to uncircumcised men. Of the 281 participants who responded to this question, 34.2% stated “less”, 10.3%—“more”, 37.4%—“about the same”, and 18.1%—“don’t know”. Answers to this question were correlated to questions about HIV/AIDS knowledge (Kendall’s tau-b coefficient r = 0.15, *p* < 0.0002).

### 4.7. Willingness to Recommend MC to Adults at Risk of Contracting HIV

Of 305/539 respondents who answered this question (56.6%), 167 (54.8%) declared they would recommend MC to adults at risk of contracting HIV, 78 (25.6%) would not, and 60 (19.7%) were not sure.

### 4.8. Factors Associated with Students Willingness to Recommend Adult MC 

Table 3 demonstrates bivariate analyses of determinants for the willingness among final year medical students to recommend MC to adults at high risk for HIV. Poles were on average more willing to discuss (60.9% vs. 41.8%; *p* = 0.003). Among those who believed that circumcised men felt more intense sexual pleasure, more were willing to discuss MC (73.2% vs. 52.2%; *p* = 0.03). Among students with knowledge, MC reduces HIV risk, and more were willing to discuss it with the patient (60.8% vs. 39.0%; *p* = 0.001). Among students familiar with knowledge on MC ex re HIV risk reduction, more were willing to discuss MC (60.8% vs. 39.0%; *p* = 0.001).

**Figure 1:**
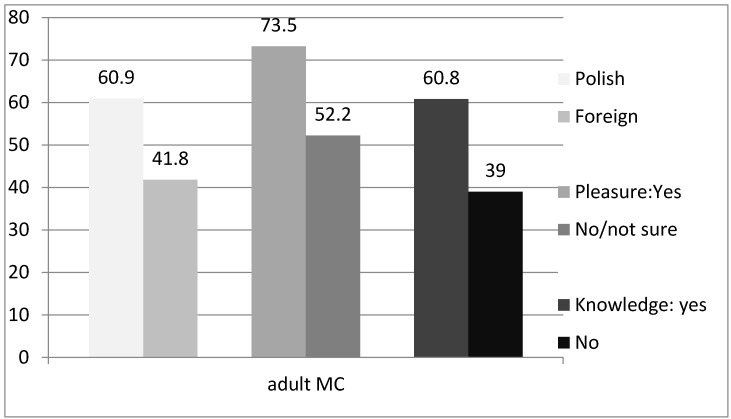
Willingness to recommend male circumcision (MC) to adults at high risk for HIV by nationality, belief that circumcised men experience more sexual pleasure, and knowledge about HIV/AIDS.

Statistically significant differences between selected determinants are presented in Figure 1.

Regression analyses were performed to assess the factors associated with a willingness of the students to recommend adult MC. Those aware that MC reduces HIV risk, as well as those who believed that circumcised men feel more intense sexual pleasure were more willing to recommend the procedure (OR = 2.13 and OR = 3.35, respectively). There was no significant association between student willingness to propose MC and: age, gender, religion, being circumcised, knowing someone who is circumcised, belief that MC is safe or that it increases penis sensitivity, and HIV/AIDS knowledge (Table 4). 

## 5. Discussion

### 5.1. Overview of the Results

One in six male respondents was circumcised, and the rate was nine times higher among foreign students as compared to Polish students. Although overall HIV/AIDS knowledge was high, only about one-third of participants knew that MC reduces the chance of HIV infection. More than half of the respondents declared that they would recommend MC for adult patients with HIV infection risk. Regression analysis revealed that personal belief that circumcised men felt more intense sexual pleasure and knowledge that MC reduces HIV risk were predictors of a willingness to recommend adult MC. 

### 5.2. Male Circumcision Rates

According to most recent estimations, 37–39% of men are circumcised globally; with approximately half of these procedures being carried out for religious/cultural reasons [9,19]. Morris et al. [19] estimated that MC prevalence rates in Europe vary between 0.1–48%, noting higher figures for countries in which males are circumcised for religious reasons. In Poland, where MC is performed mainly to treat adverse medical conditions, the estimated MC prevalence rate is 0.11%, which is the lowest in the EU. However, that estimation is regarded as conservative by the authors. According to our results, about 5% of men were circumcised; all for medical reasons. This is in line with the results of a survey conducted in Denmark (4.5%), where non-medical circumcision is rare [20]. MC prevalence rates estimated for other Central European countries vary from very low (Czech Republic, Slovakia, Hungary) to moderately low (Austria, Slovenia, Germany), with the same being true for Scandinavia (3.0–5.3%) [19]. High MC prevalence rates observed among foreign students can be explained by a higher number of circumcisions performed for religious reasons.

### 5.3. Knowledge of MC Health Benefits Regarding HIV Infection

We found that knowledge of HIV/AIDS among final year medical students was high (around two-thirds scored higher than 75% in knowledge level scores). Students gained information about MC from credible sources, including university staff and healthcare personnel. Despite this, most lacked the knowledge of MC and its health benefits regarding HIV infection. Only a third knew that circumcised men were less likely to become infected with HIV, which is alarming, given that prospective doctors should be knowledgeable on the potential benefits of MC. 

The reason for this gap in knowledge might be that, although the issue of MC is well covered by the medical curriculum, the procedure is usually discussed only when studying urology, and therefore students are taught mainly about its medical indications, performance, and complications, rather than its health benefits regarding HIV. A similar lack of knowledge among medical staff was reported previously [21,22,23]. For instance, only 23% of health care providers in the United States correctly answered that MC could reduce HIV acquisition by 60% [21].

### 5.4. Willingness to Recommend Adult MC 

This study also illustrates that for medical students, MC is an area that seems to have cultural sensitivity. We have experienced challenges when tackling this subject and the response rate to questions about the willingness to discuss adult MC was surprisingly low. 

In our study, females were less willing to recommend MC, although the differences were not statistically significant. This is in line with the results of a study conducted by Carbery et al. [21], who found that a relatively large proportion of American female physicians were not comfortable counseling patients on MC. Many reported they did not understand the benefits and risks well enough to counsel adult men or parents of newborns (one-fourth and more than a half, respectively). The authors suggest that one of the reasons for this gender disparity might be that males identify with the procedure to a greater extent, as a significantly higher percentage of MCs are performed by male physicians. 

Around 35–40% of the students in our study were unsure if MC would increase or decrease sexual pleasure and penis sensitivity, which was also found in other research [23]. So far, studies have offered conflicting results regarding MC and sexual pleasure or penile sensitivity [1,24,25,26]. However, current high-quality surveys suggest that MC has no adverse effect on sexual function, sensitivity, sexual sensation or satisfaction [24,26]. This study suggests that personal beliefs influence MC acceptability among future practitioners. The willingness to recommend adult MC was much higher among students who believed that circumcised men felt more intense sexual pleasure. These results do not correspond to AAP recommendations, that physicians should explain the benefits and risks of MC in a nonbiased manner [6]. Personal beliefs as determinants of MC acceptance among students could be explained by using the Health Belief Model, in which various psychological factors are to be believed to influence an individual’s decision regarding a beneficial health action [27]. The results of this study show that an individual’s assessment of the value of engaging in MC promotion is not necessarily based on its effects in decreasing the risk of HIV transmission, but rather effects which may influence sexual functioning. Probability analysis showed that better knowledge regarding the effect of MC on sexual pleasure might increase the acceptability of MC among healthcare providers [23]. 

Sahay et al. [28] identified religion as a cohesive force within circumcising (CC) and non-circumcising communities (NCC) in India. According to the authors, non-medical MC is a religious faith based ritual, which results in cohesion and bonding within the practicing community, but also in polarization with opposing views between CC and NCC. However, religion did not emerge as an important determinant of student willingness to counsel on adult MC. Thus, it can be concluded that in northern Europe and Poland, religious practice may not play a role in influencing decisions made by prospective doctors regarding this issue. Even though MC is universally associated with Islam and Judaism and could face challenges in its acceptability among other religious followers, most other religions currently maintain a neutral position on the practice of non-religious circumcision [9,29]. 

Regarding the HIV epidemic in Europe, the males are the more affected gender [11]. Voluntary non-medical adult MC can make a contribution to combination prevention due to its unique features: it is a single event without ongoing adherence challenges that has lifetime direct benefits [30,31]. However, assuming that adult MC might be promoted at a national level in traditionally non-circumcising countries, several issues should be taken into consideration. Firstly, evidence that MC reduces the risk of contracting HIV infection should be thoroughly studied among countries and also among subgroups, which vary from those reported in RCTs by socioeconomic status, culture, and tradition. It would also be valuable to consider evidence from subgroups that vary in demographics or sexual orientations. 

A literature review of the impact of MC on HIV-related issues is mainly based on RCTs conducted among adult men in Africa [1], which revealed that MC reduces the risk of infection with HIV and some STIs in the settings of high HIV and STIs (Sexually Transmitted Infections) endemicity; post-trial follow-up demonstrated that the efficacy of MC to reduce HIV acquisition increased even further over time [9]. Further support has been provided by meta-analyses [32], effectiveness studies in the implementation of MC [33] and biological evidence [34]. The African trials results also appear to be relevant to heterosexuals at high risk of STIs in countries with the highest levels of socioeconomic development, as well as among different cultures and traditions [9]. Observational studies in the United States involving circumcised heterosexual men have found the protective medical effects of MC consistent with those found in the African trials. For instance, in a study among men with known heterosexual HIV exposure visiting an STI clinic in Baltimore, Maryland, HIV prevalence among uncircumcised men was 22%, but only 10% among circumcised men [35]. A recent study by Chemtob et al. has provided much needed evidence demonstrating that MC was associated with reduced HIV acquisition in heterosexuals in Israel—the country in which HIV prevalence is low [36]. 

Socioeconomic factors may influence circumcision prevalence, especially in countries with a more recent uptake of the practice. Studies conducted in England, the United States, and Australia found that the rate of MC was associated with higher levels of education and income. Low circumcision prevalence was observed among recent immigrants, many of whom were more likely to be of lower socioeconomic status [37]. Further studies from traditionally non-circumcising European countries are needed to better assess this issue. 

Regarding other sexual orientations, it is unknown whether MC prevents HIV acquisition in MSM, although there might be a protective effect for men who engage mainly in insertive anal intercourse [1]. The International Antiviral Society-USA suggests that circumcision be discussed with MSM who primarily engage in insertive anal sex, especially in areas where HIV is common [38]. Existing work suggests that MC for MSM should reach its maximum potential in settings where HIV incidence among MSM is high, reported willingness for prophylactic circumcision is high, and pre-existing circumcision rates are low [39]. Further analysis is needed to properly address this challenging issue in the European region. The potential impact and cost-effectiveness of voluntary MC are not uniform and vary by age of circumcised males [40]. Therefore, studies based on mathematical models should be initiated to help examine the potential effects on programme impact and the cost-effectiveness of prioritizing specific subpopulations by age [30,40]. 

It is important that prospective doctors have the knowledge needed to counsel adult men being at risk of HIV infection. Given the observed lack of knowledge among students about MC health benefits, it could be concluded that one of the main barriers to scaling up the procedure identified in this study, might be the healthcare professionals themselves. Having adequate knowledge was significantly associated with student support for adult MC, which is in line with the results of a previous study carried out among physicians [21]. 

## 6. Limitations

There were several potential limitations to the study. Firstly, the response rate regarding outcome variables was less than 100%, which may have resulted in a response bias. Secondly, not all of the relevant variables may have been measured; for instance, to study participant views on patient anxiety regarding the procedure would be of value. Furthermore, the sample is drawn from one medical university, and therefore, limits the generalizability outside of the studied population. The study presents hypothetical conditions that might not guarantee that students would be willing to propose MC in real life circumstances. However, the strength of the study lays in its pioneering character and the diversity of its respondents, who spanned multiple European countries, with different cultures and religions.

## 7. Conclusions

Any strategy that reduces HIV infection risk should be recommended. There is still room for further investigation of the potential impact and cost-effectiveness of voluntary adult MC in countries with low HIV prevalence. Still, independent studies have so far demonstrated considerable benefits of MC regarding heterosexual HIV prevention and a protective effect among MSM who predominantly practice insertive anal intercourse [1]. While voluntary MC should attract little opposition in developed countries that enjoy a cultural or religious tradition of MC [31,35,36], it might be met with skepticism in some European countries where cultural bias against MC persists, also among health care professionals [10,26,41].

This study has disclosed challenges in implementing this prevention modality in the North European region and Poland. More than half of the final year medical students declared that they would recommend MC for adult patients at HIV risk. It is a significant finding, given that physicians are often perceived as a credible source of information when making decisions about health care [21]; this refers also to MC [28]. 

Although medical students presented a good level of HIV/AIDS knowledge overall, they seem to be poorly trained in the benefits of MC regarding HIV risk reduction. Their willingness to discuss MC was often dependent on personal beliefs rather than scientific information. Given that the knowledgeable students were more willing to discuss MC, it is vital to provide additional evidence-based information about the procedure. That should bring about an attitudinal change and help prospective physicians feel more comfortable discussing MC with adult patients at risk of HIV. Medical curriculum should be revised to encourage topics related to MC as a strategy that reduces HIV risk. 

Our study, which is a preliminary report, encourages further analyses based on prospective studies conducted among additional groups of medical professionals, such as other medical university students, physicians, and nurses, possibly with the active involvement of other countries in the region.

## Figures and Tables

**Table 1:** Demographic characteristics of the participants, Poland, 2013–2016; n = 539.

Variable	Total		Males		Females		*p* *
	N 539	% 100	n 220	% 40.8	n 319	% 59.2	
Age (Me) Range	25 22–38		25 22–35		25 22–38		0.89
Gender							
Male	220	40.8	-	-	-	-	-
Female	319	59.2	-	-	-	-	-
Nationality							
Polish	360	66.8	152	28.4	207	38.4	0.36
Other	179	33.2	68	12.4	97	20.8	0.98
Religion							
Roman Catholic	318	59.0	128	58.2	190	59.6	0.91
Other Christian	90	16.7	27	12.3	63	19.7	0.09
Muslim	24	4.5	16	7.3	8	2.5	0.02
Atheist	107	19.9	49	22.2	58	18.2	0.29
Circumcision **	192	-	32	16.7	-	-	-
Medical reasons	-	-	16	-	-	-	-
Religious reasons	-	-	16	-	-	-	-

*p* * males versus females; ** only 192/220 males answered this question.

**Table 2:** Knowledge on HIV/AIDS; final year medical students, Poland, 2013–2016; n = 539.

Statement	Correct Answer	Answer	n	(%)
HIV can be transmitted from a mother to a child	Yes	Yes No Don’t know	518 8 13	96.1 1.5 2.4
Vaginal receptive intercourse is the most risky in terms of contracting HIV infection	No	Yes No Don’t know	126 313 100	23.4 58.0 18.6
Most people with HIV show signs of being sick in the first week after infection	No	Yes No Don’t know	51 471 17	9.5 87.4 3.1
If someone is tested for HIV, a “positive blood test” means that the person is infected with HIV for life	Yes	Yes No Don’t know	385 140 14	71.4 26.0 2.6
Have you heard about anti-retroviral therapy (ART)	Yes	Yes No Not sure	511 9 19	94.8 1.7 3.5
HIV/AIDS can be cured with ART	No	Yes No Don’t know	51 446 42	9.5 82.7 7.8
Taking ART on schedule can help someone with HIV to prolong his/her life	Yes	Yes No Don’t know	482 47 10	89.4 8.7 1.9
Once a person starts ART, he/she should take ART every day for life	Yes	Yes No Don’t know	384 97 58	71.2 18.0 10.8
Missing doses of ART leads to worsening an infection	Yes	Yes No Don’t know	467 24 48	86.6 4.5 8.9
A mother who is infected with HIV can reduce the risk of giving the virus to her baby by taking certain medicines during pregnancy	Yes	Yes No Don’t know	480 24 35	89.1 4.5 6.4
Globally ART is available to almost all people who need it	No	Yes No Don’t know	240 97 202	44.5 18.0 37.5
People can reduce their chances of getting HIV by using a condom every time they have sex	Yes	Yes No Don’t know	513 17 9	95.2 3.2 1.6

**Table 3:** Bivariate analyses of determinants of final year medical students’ willingness to recommend male circumcision (MC) to adults at high risk for HIV, Poland, 2013–2016; n = 539.

Variable	n	N	%	*p*
Gender				
Male	58	92	63.0	0.07
Female	109	213	51.2	
Age				
<25	118	200	59.0	0.06
≥25	43	93	46.2	
Religion				
Atheist	29	63	46.0	0.13
Other	135	234	57.7	
Nationality				
Polish	126	207	60.9	0.003
Other	41	98	41.8	
Being circumcised				
Yes	11	13	84.6	0.18
No	43	71	60.6	
Knowing circumcised man				
Yes	40	77	51.9	0.66
no	127	228	55.7	
MC is safe				
Yes	54	106	50.9	1.0
No	7	14	50.0	
Circumcised Men experience more sexual pleasure				
Yes	25	34	73.5	0.03
No/Not sure	141	270	52.2	
MC increases the sensitivity of penis				
Yes	35	65	53.8	
No/Not sure	131	238	55.0	0.98
HIV/AIDS knowledge level				
Good	127	222	57.2	0.16
Mediocre/Poor	40	83	48.2	
MC decreases the chance to acquire HIV				
Yes	135	222	60.8	
No/Not sure	32	82	39.0	0.001

**Table 4:** Logistic regression model: association of willingness to recommend male circumcision (MC) to adults at high risk for HIV with variables selected with the use of a stepwise approach (OR’s estimates, 95% CIs of OR estimates), Poland, 2013–2016; n = 539.

Variable	OR *	CI	*p*
Knowledge on MC ex re HIV risk reduction	2.13	1.22–3.77	0.008
Belief that circumcised men get more sexual pleasure than uncircumcised	3.35	1.44–8.77	0.008

* Odds ratio = ratio between the two categories tested in each variable, controlling for other variables.
